# Antimicrobial Use Rates by Patient Care Units using NHSN Antimicrobial Use Option in TN Reporting Facilities, 2015–2023

**DOI:** 10.1017/ash.2024.148

**Published:** 2024-09-16

**Authors:** Dipen M Patel, Glodi Mutamba, Callyn Wren, Christopher Evans

**Affiliations:** Tennessee Department of Health

## Abstract

**Background:** Tracking antimicrobial use (AU) is a Core Element of Hospital Antimicrobial Stewardship Programs and important to help curb the public health threat of antimicrobial resistance. The National Healthcare Safety Network’s (NHSN) AU Option serves as a way for facilities, healthcare systems, and health departments to track and report AU rates within their jurisdictions. Many analyses at the state and national levels do not assess unit-level AU rates. This study investigates AU rates among patient care units in Tennessee reporting facilities from 2015 to 2023 and the most frequently used antimicrobial agents based on AU rates within select unit types. **Methods:** A retrospective analysis was conducted utilizing data obtained from the NHSN AU Option for inpatient units in Tennessee acute care hospitals. Units were defined as critical care (including neonatal), ward, oncology ward, stepdown, operating room (OR), and mixed acuity and specialty care areas, termed ‘other’. Unit types with fewer than five facilities represented were excluded. AU rates were determined by Antimicrobial Days of Therapy (DOT) per 1000 Days Present (DP). Statistical analyses , including descriptive statistics and comparison among the units by ANOVA test , were calculated using SAS Version 9.4. **Results:** Eighty-three facilities reported at least one month of data into the NHSN AU Option between 2015–2023. Among 70 facilities reporting inpatient units, the highest AU rate was observed in oncology ward units (n=12, 1114.6 DOT/1000 DP). A significant difference in AU rates was observed between oncology ward units compared to different unit types (p < 0 .001). Vancomycin, ceftriaxone, and piperacillin/tazobactam were the most used antimicrobials with AU rates of 83, 76, and 65 DOT/1000 DP, respectively. Vancomycin AU rates were significantly higher in oncology ward units compared to stepdown, ward, other, and OR units (p < 0 .0001). Ceftriaxone AU rate was significantly higher in stepdown units compared to oncology ward, other, and OR units (p < 0 .0001). Piperacillin/tazobactam AU rate was significantly higher in critical care units compared to different unit types (p < 0 .0001). **Conclusion:** During the study period, the AU rate varied across hospital inpatient units in Tennessee, with the highest AU rate in oncology wards. This unit-specific approach is critical to address the diverse antimicrobial prescribing behaviors observed, indicating that interventions should be customized to each unit’s distinct antimicrobial usage patterns for successful stewardship efforts. Targeted strategies focused on individual wards rather than facility-wide initiatives appear essential for effective reduction in antibiotic usage.

